# Metabolomics in viral hepatitis: advances and review

**DOI:** 10.3389/fcimb.2023.1189417

**Published:** 2023-05-17

**Authors:** Jiajia Yang, Dawei Wang, Yuancheng Li, Hongmei Wang, Qiang Hu, Ying Wang

**Affiliations:** ^1^ Department of Infection Management, The Affiliated Suzhou Hospital of Nanjing Medical University, Suzhou Municipal Hospital, Gusu School, Nanjing Medical University, Suzhou, China; ^2^ Department of Infectious Disease, The Second People’s Hospital of Yancheng City, Yancheng, China; ^3^ Institute of Dermatology, Chinese Academy of Medical Sciences and Peking Union Medical College, Jiangsu Key Laboratory of Molecular Biology for Skin Diseases and Sexually Transmitted Infections (STIs), Nanjing, China; ^4^ Department of Respiratory and Critical Care Medicine, The Affiliated Suzhou Hospital of Nanjing Medical University, Suzhou Municipal Hospital, Gusu School, Nanjing Medical University, Suzhou, China

**Keywords:** viral hepatitis, metabolomics, pathogenesis, biomarkers, treatment

## Abstract

Viral hepatitis is a major worldwide public health issue, affecting hundreds of millions of people and causing substantial morbidity and mortality. The majority of the worldwide burden of viral hepatitis is caused by five biologically unrelated hepatotropic viruses: hepatitis A virus (HAV), hepatitis B virus (HBV), hepatitis C virus (HCV), hepatitis D virus (HDV), and hepatitis E virus (HEV). Metabolomics is an emerging technology that uses qualitative and quantitative analysis of easily accessible samples to provide information of the metabolic levels of biological systems and changes in metabolic and related regulatory pathways. Alterations in glucose, lipid, and amino acid levels are involved in glycolysis, the tricarboxylic acid cycle, the pentose phosphate pathway, and amino acid metabolism. These changes in metabolites and metabolic pathways are associated with the pathogenesis and medication mechanism of viral hepatitis and related diseases. Additionally, differential metabolites can be utilized as biomarkers for diagnosis, prognosis, and therapeutic responses. In this review, we present a thorough overview of developments in metabolomics for viral hepatitis.

## Introduction

Viral hepatitis is a significant global public health issue with hundreds of millions of victims and significant morbidity and mortality rates. Hepatitis A virus (HAV), hepatitis B virus (HBV), hepatitis C virus (HCV), hepatitis D virus (HDV), and hepatitis E virus (HEV) are the five biologically unrelated hepatotropic viruses that cause the majority of viral hepatitis worldwide (Xiang et al., 2022). Although HAV does not progress into a chronic infection, HBV, HCV, HDV, and HEV occasionally do. HBV and HCV in particular show a strong correlation with the occurrence of chronic infections. HBV and HCV infections cause the majority of viral hepatitis-related deaths. The statistics indicate that 296 million individuals were estimated to have hepatitis B, 58 million to have hepatitis C, and 1.1 million died as a result of viral hepatitis infections in 2019. As the body’s primary metabolic organ, the liver will inevitably change the linked metabolic network once sickness arises, which will have an impact on the levels of many endogenous small molecule metabolites in the body. The detection of these endogenous small molecules in tissues or fluids can be used as sensitive indicators of liver injury and can aid in the analysis and understanding of the etiology and pathogenesis of liver diseases (Xiang et al., 2022; Wolfe et al., 2023).

Metabolomics is a new systems biology field that uses methods from analytical chemistry to characterize the endogenous small-molecule metabolites that are found in tissues and biofluids. A variety of biological activities, such as mitochondrial activity, lipid biosynthesis and metabolism, glucose and glutamine metabolism, and nucleotide biosynthesis, can all be simultaneously analyzed using metabolomics. As metabolites are the downstream end products of gene and protein expression, they most closely correspond to phenotype (Manchester and Anand, 2017). In recent years, through the use of metabolomics research, the pathogenic mechanisms of infectious disorders, such as coronavirus disease 2019 (COVID-19) (Hasan et al., 2021), human immunodeficiency virus (HIV) disease (Olund Villumsen et al., 2021), and herpesviruses (HPV) infection (Abudula et al., 2020) have been successfully understood. Many researchers have suggested etiology and potential biomarkers for viral hepatitis and related diseases utilizing metabolomics resources, demonstrating the promise of the metabolomics method in increasingly complicated disorders (Cui et al., 2021). Therefore, this review covers the applications of metabolomics in various forms of viral hepatitis and associated diseases to shed light on the progression of these disorders.

## Metabolomics technology

In 1971, Pauling et al. performed the first large-scale quantitative detection of hundreds of substances in various biological fluids to reflect the functional state of biological systems, ushering in a new era of metabolomics research (Cui et al., 2018). The concept of metabolomics was first proposed by Nicholson et al. in 1999 (Nicholson et al., 1999). It is a science that analyzes the changes of small molecule metabolites (relative molecular mass <1 000) in biological tissues. Metabolomics research can be divided into non-targeted and targeted analysis methods, which differ in the quantitative level of research targets, sample preparation, accuracy and precision of obtained results, and the number of identified metabolites (Dunn et al., 2011). Untargeted metabolomics enables the investigation of complex interactions between a variety of metabolites from various pathways before the formulation of hypotheses, resulting in the discovery of new metabolites but also producing large data sets and experimental challenges in overcoming the identification of previously unidentified metabolites. Targeted metabolomics involves measuring or quantifying metabolites whose pertinent chemical structure and biological activity are known. Targeted metabolomics can be used to more accurately and selectively explain how some chemicals are associated with particular disease conditions (Roberts et al., 2012).

**Figure 1:**
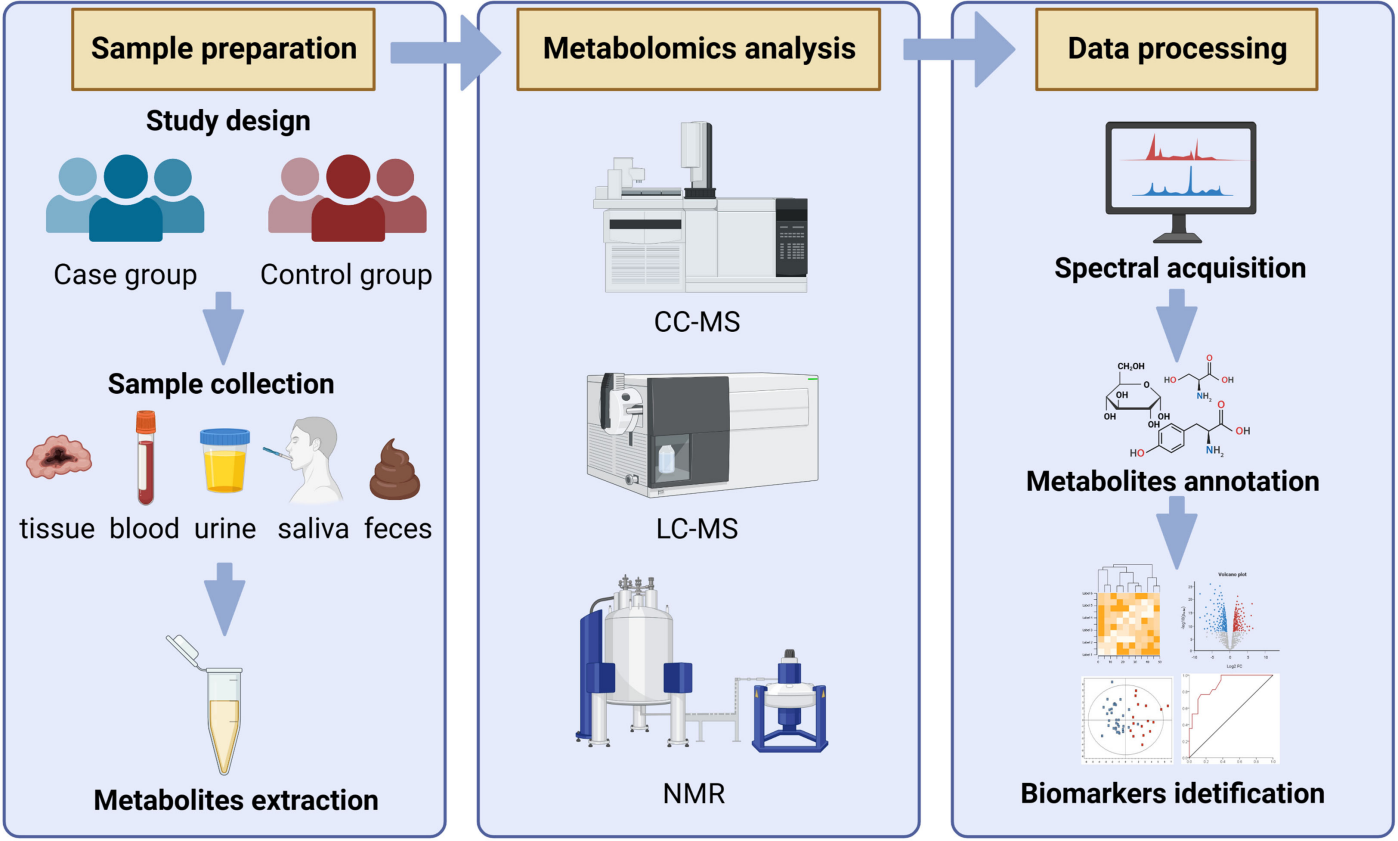
The general workflow of metabolomics analysis.

The classic metabolomics process includes the collection of samples (plasma, serum, urine, saliva, cell and tissue extracts and feces, etc.) and sample pretreatment, metabolite measurement, data processing, and statistical analysis, in order to obtain small molecules of biological significance that are closely related to metabolic characteristics (Roca et al., 2021). The commonly used measurement methods of metabolomics include nuclear magnetic resonance (NMR), liquid chromatography-mass spectrometry (LC-MS), and gas chromatography-mass spectrometry (GC-MS) (Carbone et al., 2021; Edison, 2021). NMR is simple to pretreat samples, does not damage the sample structure, requires a small number of samples, has a short measurement time, has high repeatability, can perform non-selective detection of specific metabolites, and can also classify the phenotype, but the sensitivity is not as good as MS, for some compounds with low content may not be detected. The volatility and thermal stability of the measured components are not required by LC-MS technology, and the preliminary processing is simple, allowing for rapid and efficient detection. However, there is a lack of a reference standard spectrum library, and identifying metabolites requires the use of standard substances, which is relatively difficult. GC-MS technology has a high resolution and detection sensitivity, as well as a standard spectrum library for reference, which can be used for qualitative metabolite characterization. However, derivation processing is required for samples with low volatility, and the early processing is complicated and may result in the loss of signals of some substances. As there is no single technology that can simultaneously measure and identify all metabolites in samples due to the diversity and complexity of metabolites, it is required to combine the data from different analytic platforms to gain more thorough metabolite information (Pomyen et al., 2020; Spina and Saliba, 2021). Bioanalytical procedures are used to get the raw data, which is then analyzed utilizing several stoichiometric approaches (Ghosh et al., 2020). The principal component analysis (PCA), hierarchical cluster analysis (HCA), and other unsupervised pattern recognition techniques, as well as supervised pattern recognition techniques like orthogonal partial least squares (OPLS), partial least squares-discriminant analysis (PIS-DA), and random forest analysis (RFA), are currently the most widely used pattern recognition techniques. The most often utilized of these are PCA, PLS-DA, and OPLS-DA (Trainor et al., 2017). The general workflow of metabolomics analysis was shown in **Figure 1**.

The key metabolic pathways and associated enzymes in the occurrence and development of diseases can be screened through the systematic characterization of metabolic profiles in the biological samples of subjects, and from there, the regulatory data of upstream related proteins and genes can be gathered (Schmidt et al., 2021). A succession of biological events in the entire biomolecular network during the diseased process can thus be understood by individual metabolomics analysis (Alarcon-Barrera et al., 2022). In terms of disease diagnosis, prognosis, drug effects, and knowledge of disease pathogenesis and progression, metabolomics has special advantages in the field of medicine.

## Metabolomics in hepatitis A and related diseases

HAV is a positive-strand RNA virus that spreads by the fecal-oral route. HAV epidemics are frequently linked to bad hygiene, crowded living conditions, or tainted food and water (Abutaleb and Kottilil, 2020). Children with HAV infections typically show no symptoms, whereas adults with HAV infections may exhibit signs such as hyperbilirubinemia, stomach discomfort, and jaundice. Hepatitis A is a self-limiting disease that primarily causes acute hepatitis and has a favorable prognosis (Xiang et al., 2022). Yamane et al. (1994) conducted phosphorus-31-31 NMR analysis on 26 patients with acute hepatitis A to assess the changes in phospholipid metabolism in hepatocytes during acute hepatitis A virus infection. Six signaling components corresponding to phosphomonoester (PME), inorganic phosphate (Pi), phosphodiester (PDE), and adenosine triphosphate isomers (α-, β-, and γ-ATP) were detected in the liver using spectroscopy in all patients and healthy subjects. Their results showed that the PME-PDE ratios in the patients significantly increased compared to the ratios in the controls during the early stages of the disease. The PME/PDE ratios reverted to controls within six weeks of the disease’s onset. According to the time course study, the PME/PDE ratio and the length of time after onset are inversely correlated.

In addition, Yamane et al. (1994) discovered that the spectral alterations shown in acute viral hepatitis A-infected human livers were comparable to those seen in rat livers that had undergone regeneration. Their findings demonstrated that metabolic profiling could provide non-invasive real-time information on human liver metabolism and could be a key method for monitoring liver regeneration (Yamane et al., 1994). At present, there are few studies on the metabolomics of hepatitis A, but this study provides a certain perspective and foundation for future research.

## Metabolomics in hepatitis B and related diseases

Globally, the hepatitis B virus (HBV) is the most frequent cause of both acute and chronic liver disorders, infecting around 4 million individuals annually, primarily in Asia and Africa (Khan et al., 2021). In about 10% of HBV-infected people, persistent infections like liver fibrosis and cirrhosis will appear (Lu et al., 2018). A million people per year pass away from chronic liver diseases due to hepatitis B. Most chronic hepatitis B (CHB) patients don’t have any noticeable symptoms at first, but as the condition worsens, they eventually get cirrhosis of the liver and hepatocellular carcinoma (HCC) (Su et al., 2022). The development of metabolomics investigations has sped up our understanding of the pathogenesis, early diagnosis and treatment of HBV infection.

## Metabolomics in the pathogenesis of hepatitis B and related diseases

As of right now, there are no drugs or forms of treatment that will completely eradicate HBV, therefore treatment must last a lifetime (McNaughton et al., 2021; Lian et al., 2022). HBV reinfection, particularly HBV infection in extrahepatic organs, has grown to be a significant risk factor for prognosis (Yue et al., 2002). The prospective therapeutics will become more clear if the mechanisms of HBV infection-associated liver diseases (HBV-CLD) is understood.

The microbiota can produce metabolites and active ingredients that participate in the regulation of host metabolism and immunity and is closely related to the pathological processes of HBV-CLD. Infection with HBV has been linked to intestinal microbial dysregulation. By combining investigations of the fecal microbiome and metabolome, a recent study (Shen et al., 2023) revealed a striking shift in the gut microbiota and metabolites in HBV-CLD patients. The progression of the disease and antiviral therapy were identified as the two main contributors to the shift. Through fecal metabolomics technology, Li et al (Li, 2021). also found that intestinal amino acid and glucose metabolism become aberrant when the HBV infection progresses.

Additionally, oral microbiota and metabolites may be involved in the development of HBV-CLD. Zhao et al. (2018) reported that the relative compositions of the tongue coating microbiotas and metabolites in the CHB patients were significantly different from those of the healthy controls, and the number of amino acid metabolites was greater in the CHB yellow tongue coating patients than in the white tongue coating patients. The researchers inferred that the metagenomic pathways that were enriched in the CHB yellow tongue coating patients were mainly those involved in amino acid metabolism. Another study by Zhao et al. (2013) showed significant differences in 17 urinary metabolites among CHB patients with different tongue coatings, and these metabolites may be the material basis of the two tongue coatings differentiation. Compared with the CHB white tongue coating patients, the CHB yellow tongue coating patients had higher HBV-DNA titers that is considered to be the most direct etiological basis of hepatitis B virus replication and infection (Gu et al., 2009).

Serum and urine are the simplest body fluid samples to collect. The detection of metabolite levels in serum and urine can provide new insights into disease pathogenesis and progression. Yang et al. (2016) used GC-MS metabolic profiles in serum and urine samples from CHB patients to conduct a covariation analysis to investigate metabolic anomalies in CHB. According to their findings, CHB patients may have altered pathways for a) glycine metabolism and b) the bridge that joins fatty acids with the tricarboxylic acid (TCA) cycle. Simillion et al. (2017) used GC-MS to analyze the plasma and urine of groups of HBV patients and healthy controls. They discovered 10 metabolite correlations for the HBV plasma and urine, but none for the controls. Their findings showed that increased glucose uptake, glycolysis, and pentose phosphate pathway metabolism were all consistent with HBV hepatitis; the latter uses xylitol and generates threonic acid, which may also be imported by glucose transporters. Xie et al (Xie, 2016). used serum metabolomics technology to screen out the metabolites isocitrate and isocitrate and lysophosphatidyl ethanolamine with significant differences in their expression in HBV DNA positive and negative groups, suggesting that the trilboic acid cycle pathway and the phospholipid metabolism pathway may be related to the pathogenesis of hepatitis B. Based on the serum metabolic profile, the metabolic processes identified by Du et al. (2011) that are related to the occurrence and development of HBV-related cirrhosis include amino acid metabolism, phospholipid metabolism, bile acid metabolism, tribolic acid cycle, and nicoculiamine metabolism. The biological processes involved include energy metabolism, protein synthesis and degradation, cell necrosis, inflammatory response, neurotransmitter synthesis, and degradation, etc. It can be seen that the metabolic abnormalities of liver cirrhosis are involved in all the main biochemical processes, among which amino acid metabolism is the most obvious. This may be because amino acids are widely involved in proteins, energy, and other processes in the body, most of which take place in the liver.

Gao et al (Gao, 2016). performed a paired comparative analysis of the metabolome of liver cancer tissue, paracancer tissue, and distant paracancer tissue from 39 patients with liver cancer, revealing that in order to balance the energy demands of rapid growth and proliferation of liver cancer cells as well as the material demands for biomacromolecule synthesis, on the one hand, glucose intake was increased and the glycolysis pathway was activated, while the tridellic acid pathway was deactivated. On the other hand, it significantly activates the pentose phosphate pathway (PPP), which, in addition to providing energy, primarily provides a variety of raw materials for anabolic metabolism and also produces a large amount of NADPH as a reducing power for biosynthesis and antioxidant stress damage. Furthermore, it increases glutamiculamine metabolism. Glutamiculamine is another non-mitochondrial ATP energy source for liver cancer cells, and it can provide energy and biomacromolecular synthesis raw materials for tumor cell growth and proliferation, as well as help maintain tumor cell microenvironment homeostasis. Furthermore, it improved the oxidative utilization of fatty acids and amino acid metabolism, providing enough energy for the growth and proliferation of liver cancer cells. By using untargeted NMR spectroscopy, Lan et al (2022). found that HBV-transgenic mice had significantly different metabolites from their wild-type littermates in the heart, liver, spleen, lung, kidney, pancreas, and intestine. They also discovered several metabolites, including branched-chain amino acids (BCAAs), a general potential biomarker in HBV-transgenic mice, which showed an increasing trend in most tissues, and choline, which may be a liver-specific biomarker for HBV detection. In HBV transgenic mice, the elevated choline concentrations may be associated with biofilm synthesis and lipid metabolism, providing lecithin for HBV replication. In addition, in response to oxidative stress caused by HBV replication, in the absence of glutathione, the concentration of BCAAs is increased to produce more alternative antioxidants.

HepG2.2.15 cells have a similar phenotype to HBV infected liver cells and have become a common model for the study of HBV infection (Wang et al., 2009). Through NMR spectroscopy, Li et al. (2015) conducted NMR metabolomics to detect and analyze metabolome differences between HepG2 and HepG2.2.15 cells, and found that HBV infection upregates hexoamine and phosphatidylcholine synthesis in host cells, generates oxidative stress and promotes central carbon metabolism and nucleoside synthesis. Using NMR metabolomics spectroscopy, Wan et al. (2017) discovered that HBV infection enhanced glycolysis, promoted cellular uptake of glucose, and improved the pentose phosphate and hexosamine pathways for the production of RNA, DNA, and nucleotide sugars to promote HBV replication. Furthermore, Li et al. (2017) confirmed through GC-MS analysis that HBV infection can alter the metabolism of amino acids and lipids in host cells, which is connected to numerous metabolic pathways including glycolysis, fatty acid metabolism, and amino acid metabolism. In addition, the information of metabolites obtained by GC-MS analysis is a good complement to that obtained by NMR. Recently, Hu et al. (2021) performed a metabolomics experiment via LC-MS, which demonstrated that host antiviral reaction to HBV is positively regulated by the hexosamine biosynthesis pathway by enhancing O-GlcNAc transferase-mediated protein O-GlcNAcylation *in vitro* and *in vivo*. Hepatitis B virus X protein (HBx) plays an important role in HBV-related HCC. To thoroughly research how HBx affects cell metabolism, Yue et al (Dan et al., 2016). used an NMR-based metabolomics approach. The findings suggested that HBx causes DNA damage at first, affects nucleic acid metabolism later, and then prevents DNA repair, leading to the development of HCC. Taking into account of the above evidence, metabolomics can be applied to explore the pathogenesis and progression of hepatitis B and related diseases.

## Metabolomics in identifying biomarkers of hepatitis B and related diseases

Prolonged HBV infection may lead to chronic hepatitis, liver fibrosis, cirrhosis, and hepatocyte (HCC), and some patients may progress to liver failure, which seriously endangers the health and quality of life of patients. Therefore, early diagnosis and grading evaluation of hepatitis B and related diseases are of great significance (**Table 1**).

**Table 1:** Metabolites as the diagnostic biomarkers of hepatitis B and related diseases.

The first author	Year	Cases	Sample type	Platform	Up-regulated metabolites	Down-regulated metabolites
Nguyen et al (2021)	2021	CHB vs. healthy controls	serum	NMR	glutamate, succinate, acetate, acetone, and formate	methionine, glutamine, asparagine, pyruvate, glycine, lactate, valine, creatinine and alanine
Gilany et al. (2019)	2019	CHB vs. healthy controls	saliva	NMR	propionic acid, putrescine, aceticacid, succinic acid, tyrosine	L-lacticacid, butyric acid, pyruvate, 4-pyridoxic acid, 4-hydroxybenzoic acid
Hou et al. (2015)	2015	hepatitis B vs. healthy controls	serum	GC-MS	citric acid, 5-oxoproline, N,N-dimethylglycine, malonic acid, aconitic acid, pyruvic acid	aminooxyacetic acid, glucose, glutamine, tyrosine
Zhang et al. (2013)	2013	HBV cases vs. healthy controls	urine	UPLC- MS	tyrosinamide, biotin sulfone, hexanoic acid, 1-aminonaphthalene, 7-dehydrocholesterol, azelaic acid	alpha-n-phenylacetyl-l-glutamine, 5-oxo-heneicosanoic acid, D-glucosaminide, phenyl sulfate, 2-methylhippuric acid
Dittharot et al. (2018)	2018	CHB vs. healthy controls	urine	GC/MS	palmitic acid, stearic acid, oleic acid, benzoic acid, butanoic acid, cholesterol, glycine, 3-heptanone, 4-heptanone, hexanal, 1-tetradecanol and naphthalene	
Zhang et al. (2010)	2010	liver failure vs. healthy controls	plasma	LC–MS	1-Linoleoylglycerophosphocholine	
Mao et al. (2008)	2008	liver failure vs. healthy controls	serum	GC-MS	glyceric acid, cis-aconitic acid, citric acid	
Nie et al. (2014)	2014	ACLF vs. LC	serum	UPLC/MS	lysophosphatidic acid,	lysophosphatidylcholine, phosphatidic acid
Yu et al. (2007)	2007	liver failure vs. normal subjects	serum	GC-MS	L-Threonine, Glycine, L-Serine, L-ThreonineProline, L-Proline, L-Serine, L-Proline, Glutamine, L-Phenylalanine, Hexadecanoic acid	3-Hydroxyl-l,2,3-propanetricarboxylic acid, 2-Deoxy-galactopyranose, D-Fructose, D-Glucose
Xue et al. (2009)	2009	cirrhosis vs. non-cirrhosis	serum	GC-MS	Acetic acid, Hexanoic acid, 1-Naphthalenamine, Butanoic acid	Sorbitol, D-Lactic acid, Phosphoric acid, D-glucitol, Glucose
Du et al. (2011)	2011	CHB vs. healthy controls	serum	LC–MS	Cysteine,glycochenodeoxycholic acid, tyramine, glycine, L-alanine, aminoadipic acid, taurine, glycine, aminoacetone, pyruvic acid	Lysophosphatidylcholine, DL-methionine sulfoxide, L-serine
Wang et al. (2012)	2012	LC vs. health controls	urine	UPLC-MS and GC-MS	Proline, Citrate, Aconitate, 2-Pentendioate, Acetyl citrate, 3,4-Dihydroxyphenylacetate, 4-Hydroxy-benzenepropanedioate, 3-Methoxy-4-Hydroxyphenylglycol sulfate, Canavaninosuccinate, Glycocholic acid, 3-glucuronide, Taurohyocholate, Glycocholate, Glycoursodeoxycholate	4-Pyridinecarboxylate Threonine, Hippurate, 2-Aminobutyrate, cis-Aconitate, Pyroglutamate, OPhosphotyrosine, Alpha-Hydroxyisobutyrate, 3-Hydroxyisovalerate, DopaxanthinAlpha-Hydroxyhippurate, L-Aspartyl-4-phosphateIsoxanthopterin, Tyrosine-betaxanthin, Estrone, Cortolone-3-glucuronide, Tetrahydroaldosterone-3-glucuronide, 11-beta-Hydroxyandrosterone-3glucuronide, N-Acetyl-leukotriene E4, 11-Oxo-androsterone glucuronide, Dehydroepiandrosterone 3-glucuronide, Androsterone sulfate, Testosterone sulfate, Androsterone glucuronide, 17-hydroxyandrostane-3-glucuronide, Glycolithocholate 3-sulfate
Qi et al. (2012)	2012	HBV-infected cirrhosis vs. alcoholic cirrhosis	serum	^1^H NMR	Creatine, Isobutyrate	Acetoacetate, Glutamine, Glutamate
Lian et al. (2011)	2011	HBV-infected cirrhosis vs. alcoholic cirrhosis	serum	LC-MS	glycocholic acid, glycochenodeoxycholic acid, hypoxanthine, stearamide	Lysophosphatidyl cholines
Cox et al. (2016)	2016	HCC vs. LC	urine	NMR	Carnitine	creatinine, hippurate, trimethylamine- N -oxide
Shariff et al. (2011)	2011	HCC vs. healthy controls	urine	NMR	Creatine, Carnitine	Glycine, trimethylamine- N -oxide, Hippurate, Citrate, Creatinine
Gao (2011)	2011	HCC vs. healthy controls	urine	HPLC/MS	Tyrosine, Citric acid, Aconitic acid, Glycochenodeoxyc holate-3-sulfate, 4-oxo-Retinoic acid, Glycoursodeoxycholic acid, 1,3-Dimethyluracil	L-gamma-glutamyl-L-1eucine, Deoxycholic acid 3-glucuronide, 3a, 7b, 12a-Trihydro xyoxocholanyl-Glycine
Sheng (2008)	2008		serum	GC/MS UPLC/MS	Glycine, valine, fructose, glucose, glucose-2-deoxypyrine	Propionic acid, L-proline
Zhang et al. (2019)	2019	HCC vs. LC	serum	HPLC/MS	L-Aspartyl-4-phosphate, 2-Aminomuconic acid semialdehyde	Lysophosphatidylcholine
Yang (2015)	2015	HCC vs. LC	serum	^1^H NMR	Leucine, lactic acid, alanine, choline, glycine	Taurine, formic acid

CHB, chronic Hepatitis B; ACLF, acute-on-chronic liver failure; LC, liver cirrhosis; HCC, hepatocellular carcinoma; NMR, nuclear magnetic resonance; GC-MS, gas chromatography-mass spectrometry; UPLC-MS, ultra performance liquid chromatography-mass spectrometry; LC-MS, liquid chromatography-mass spectrometry; HPLC/MS, high performance liquid chromatography- mass spectrometry.

Metabolomics can be used to distinguish CHB patients from healthy controls and to differentiate between the various phases of CHB. Nguyen et al (2021) analyzed and compared the serum metabolites of patients at different stages of CHB and comparing them to a healthy individual, and found that perturbations in ammonia detoxification, glutamine and glutamate metabolism, methionine metabolism, dysregulation of branched-chain amino acids, and the TCA cycle are the main factors involved in the progression of the disease, and fluctuations increasing in aspartate, glutamate, glutamine, and methionine are fingerprints of progression. Their findings revealed the effectiveness of the metabolomics approach in detecting CHB transitioning from the immune tolerance to the immune clearance phase and can provide a more detailed decision basis for starting medical treatment. Hou et al. (2015) reported significant differences in serum metabolites between HBV patients and healthy controls. The area under the curve (AUC) of the total metabolite variables consisting of citric acid, aconitonic acid, glutamine, N,N- dimethyl glycine, malonic acid in the diagnosis of HBV infections was 0.975, which is of high diagnostic value. Gao et al. (2015) found that serum metabolites alanine, malic acid, and 5-methoxytryptamine could accurately distinguish the CHB group from the healthy control group. Zhang et al (Zhang et al., 2013). conducted metabolomics analysis of urine samples from HBV-infected patients and healthy controls, and observed that biotin sulfone, 5-oxo-heneicosanoic acid, d-Glucosaminide, and 2-methylhippuric acid were significantly different in the HBV patients and controls. Additionally, the ROC curve study of these four marker metabolites produced an AUC of 0.807, which was higher than that of ALT (0.619), AST (0.624), and LB (0.628), respectively. The unsupervised grouping further demonstrated that we can distinguish between controls and HBV cases by using the metabolites as biomarkers. Dittharot et al. (2018) also reported that GC/MS-based urine metabolomics in combination with multivariate statistical analysis distinguished CHB patients from healthy controls with excellent sensitivity (95%) and specificity (85%). Using NMR technology, Gilany et al. (2019) confirmed that salivary metabolites including propionic acid, putrescine, acetic acid, succini cacid, tyrosine, lactic acid, butyric acid, pyruvic acid, 4-pyridoxic acid, and 4-hydroxybenzoic acid were different between CHB patients and healthy subjects, which can be used as markers for the diagnosis of CHB.

Hepatitis B cirrhosis is a serious and irreversible liver disease. It is very important to accurately judge the severity and development trend of the disease and carry out targeted treatment to effectively control the development of the disease. In recent years, many scholars have used metabolomics to differentiate, grade, and forecast HBV-associated cirrhosis. Seven oxylipins derived from omega-6 were discovered to be altered in patients with HBV-related liver diseases, according to the research by Lu et al (Lu et al., 2018). These seven different oxylipins, when combined with AFP, age, and sex, greatly increased the prediction of HBV-related cirrhosis. The research by Xue et al. (2009) demonstrated a distinction in serum metabolites between HBV-infected non-cirrhosis and cirrhosis patients and verified the viability of GC-MS technology for the selection of biomarkers for cirrhosis after hepatitis B virus infection.

At present, the Child-Pugh grading system is commonly used to indicate the status of liver reserve function to judge the prognosis of chronic liver disease. However, there are still some limitations in the practical application, such as the subjective interference of score calculation is strong, and the response is not sensitive enough. Du et al. (2011) found that clinical stages of hepatitis B cirrhosis can be identified using serum metabolic profiles with high accuracy. Another study (Wang et al., 2012) revealed six urinary metabolites (α-hydroxy hippurate, tyrosine-betaxanthin, 3-hydroxy isovalerate, canavaninosuccinate, estrone, and glycoursodeoxycholate) were significantly altered among cirrhotic patients with CP A, B, and C, and demonstrated the diagnostic and prognostic potential for the development of cirrhosis.

The etiology of cirrhosis is various, and some scholars distinguished the etiology by metabolomics. Qi et al. (2012) detected serum metabolites of 21 patients with hepatitis B cirrhosis, 20 patients with alcoholic cirrhosis, and 20 healthy controls by the NMR method. The results showed that creatine, acetoacetic acid, isobutyrate, glutamine, and glutamate were significantly different between patients with hepatitis B cirrhosis and patients with alcoholic cirrhosis, and could be used as specific markers for the diagnosis of the two diseases. In the study of Lian et al. (2011), oleamide and myristamide were elevated in the serum of patients with alcoholic cirrhosis but reduced in those with HBV-induced cirrhosis, suggesting that they may be useful biomarkers for differentiating between these two types of cirrhosis.

HCC usually develops gradually, with an insidious start, late onset of clinical symptoms, and a high mortality rate. Early detection, diagnosis, and therapy are therefore essential for HCC patients. HCC is clinically identified through tissue-based histopathological results, distinctive radiologic characteristics, or blood-based assays like the AFP test. It would seem that the sensitivity and specificity of such clinically applied methodologies may not be sufficient in detecting early-stage HCC, greatly decreasing the reliability. Metabolomics has recently grown in prominence in the field of oncology due to the possibility of detecting homeostasis perturbations associated with the emergence of cancer (Lee et al., 2020). Through urinary nuclear magnetic resonance spectroscopy, Cox et al. (2016) studied patients with hepatitis B hepatocellular carcinoma in Bangladesh, and the results showed that carnitine was significantly increased in HCC, and creatinine, hippurate, and trimethylamine-N-oxide were significantly reduced in HCC compared to CHB, HBV-related cirrhosis, and healthy controls. The results may help in the creation of a low-cost HCC urinary dipstick screening test. Shariff et al. (2011) explored the urinary metabolic biomarkers of HBV-related HCC in an Egyptian Population. The discriminatory metabolites they found included glycine, trimethylamine-N-oxide, hippurate, citrate, creatinine, creatine, and carnitine. The sensitivity and specificity of the technique for distinguishing patients with HCC from healthy controls and patients with cirrhosis were 100%/94% and 81%/71%, respectively. In the Chinese population, Gao et al (Gao, 2011). also showed the usefulness of urine metabolomics techniques in the differential diagnosis of HBV-related HCC from cirrhosis and normal subjects. Moreover, there are numerous studies (Sheng, 2008; Zhang et al., 2013; Huang, 2014; Gao et al., 2015; Yang, 2015; Zhang et al., 2019) demonstrated that serum metabolites can distinguish HBV-related HCC from normal subjects. Additionally, a recent study (Lee et al., 2020) on the comprehensive metabolomic profile revealed that HCC with normal alpha-fetoprotein had serum levels of O-acetylcarnitine that were significantly higher than those with higher levels of AFP, and HCC with microscopic vascular invasion (VI) had preoperative serum levels of formate that were significantly higher than those with HCC without microscopic VI. Therefore, serum metabolites could be used to help with the early diagnosis of HCC patients who tested negative for AFP and the identification of microvascular invasion to aid in preoperative surgical planning and postoperative follow-up.

As HBV-related liver failure is a serious condition with a high mortality rate, early detection, precise diagnosis, and prompt treatment are crucial. Hyperbilirubinemia and a prolonged clotting time are the main criteria used in clinical practice to diagnose liver failure. The aforementioned two indices, however, do not accurately reflect liver function, which has limitations for the early detection of the liver failure. The early diagnosis of liver failure can significantly benefit from metabolomics. By using the PCA method to analyze the raw data (roughly 1000 compounds) from the plasma of patients with HBV-related liver failure and healthy controls, Zhang et al. (2010) found that a pattern recognition profile that distinguished significantly between patients with liver failure and healthy controls could be created. The ability of this model to predict liver failure has a 94.3 percent specificity and a 100 percent sensitivity, respectively. Mao et al. (2008) used GC/MS technology to perform serum metabolomics studies on 24 patients with HBV-related liver failure and 23 healthy controls, and found that glycolic acid, cisolmonic acid, and citric acid could be used as markers for the diagnosis of liver failure. Nie et al. (2014) discovered that the HBV-related acute-on-chronic failure (ACLF) group had substantially lower levels of lysophosphatidic acid and lecithin compared to the CHB group, the hepatitis B cirrhosis group, and the normal control group. The levels of lysophosphatidyllecithin, lysophosphatidic acid, and phosphatidic acid were consistent with the severity of liver disease and could be used as potential markers to forecast the outcome of acute-on-chronic liver failure. Yu et al. (2007) observed that the amino acid and glucose chromatograms in the HBV-induced liver failure group were significantly different from those in healthy people and that the identification of amino acid and glucose chromatograms could be used to distinguish early liver failure patients. Sheng et al (Sheng, 2008). also showed that serum metabolites can distinguish hepatitis B-associated liver failure from normal subjects. Li et al (Li, 2014). found that serum metabolite profiles of hepatitis B-related ACLF and chronic liver failure (CLF) were significantly different, and linocarnitine could be used as a specific biomarker for the diagnosis of ACLF and CLF. Another study showed that when compared to the group with a poor prognosis, the group with an improved prognosis had substantially higher levels of arginine and short- and long-chain coolyl carnitine, which were potential indicators of liver failure prognosis (Gao, 2019). The artificial liver support system (ALSS) is essential for the treatment of ACLF. Nowadays, several studies have shown that serum and urine metabolomics approaches could examine the dynamic change process of biomarkers before and after ALSS treatment, and some metabolites can be utilized as indicators to evaluate the prognosis of liver failure (Guo, 2011; Hao, 2011; Huang et al., 2021). These findings indicate that the metabolites in serum, urine and saliva can be used as markers for the diagnosis, development and treatment of hepatitis B and related diseases.

## Metabolomics in understanding the drug action mechanism of hepatitis B and related diseases

Some Chinese medicines have been used to treat hepatitis B, but the mechanism of action is unclear. Metabolomics is helpful to elucidate the efficacy and mechanism of TCM. Using the methods of epigenetics and metabolomics, Zhang et al. (2022) showed that the action mechanism of *Chrysanthemi indici C* (CIC) against HBV may be the synergistic action of multiple pathways and pathways multiple targets, including related inflammatory pathways, immune pathways and lipid metabolism, through regulating epigenetic expression balance and restoring the balance of cell microenvironment. Chai et al. (2018) confirmed that Tonkinensis may mainly act on 16 target proteins to exert anti-HBV effects, and its mechanism may be related to the regulation of retinol metabolism, peroxisome proliferator activate-receptors (PPAR) signaling pathway, transcription disorders in cancer and other processes, so as to control HBV replication and regulate immune and metabolic disorders in the body. Zheng et al. (2022) found that Kushen Wumei Decoction can resist HBV. Metabolomics and network pharmacology found that the mechanism of its action of anti-HBV may be related to the regulation of sphingolipid metabolism pathway, the biosynthesis of phenylalanine, tyrosine and tryptophan, and phenylalanine metabolism pathway, and PI3K-Akt signaling pathway. Recently, the results of the LC-MS/MS analysis revealed that *C. nutans* significantly upregulated the HBV mouse markers hippuric acid, L-histidine, trehalose, D-threitol, and stachyose while significantly downregulating uridine 5’-diphosphate, cholic acid, trimethylamine N-oxide CDP-ethanolamine, and phosphorylcholine. The correlation analysis showed that C nutans modulates essential bacteria (Alistipes), exhibiting specific anti-inflammatory effects on the levels of associated metabolites hippuric acid and cholic acid. The therapeutic potential for anti-HBV infection was demonstrated by these findings, which revealed that C. nutans exhibits protective effects in HBV model mice (Zhang et al.,2023). Hence, metabolomics plays an important role in exploring the mechanism of drug therapy for hepatitis B and related diseases.

## Metabolomics in hepatitis C and related diseases

HCV virions are spherical and are single-stranded positive-stranded RNA viruses. More than 71 million people globally are affected by HCV. HCV infection is an significant contributing factor to end-disease. Chronic HCV (CHC) infection is related to advanced liver disease and can cause hepatocellular carcinoma, which generates a variety of extrahepatic symptoms.

## Metabolomics in the pathogenesis of hepatitis C and related diseases

The majority of people with HCV infection (around 85%) acquire chronic infections. As with other types of chronic liver diseases, HCV infections are accompanied by liver fibrosis. If treatment is not timely, it can even develop into HCC, which seriously endangers the life and health of patients. A MS-based lipidomic investigation of 30 patients with chronic HCV infection and 30 healthy blood donor controls was undertaken, and identified 34 downregulated metabolites and 21 upregulated metabolites. Anandamide and eight fatty acid amides were elevated, which probably stimulating the cannabinoid receptor GPR55, a crucial host component for HCV replication. In addition, several lysophosphatidylinositols, which are necessary for the formation of phosphatidylinositol 4-phosphate pools required for HCV replication and can also activate the GPR55 receptor, were markedly downregulated (Beyoğlu et al., 2023). Ma et al. (2018) demonstrated that the changes in core fucosylation are protein and site specific during the progression of fibrotic liver disease and independent of the changes in the quantity of N-glycoproteins. Simillion et al. (2017) used GC-MS to examine the plasma and urine of groups of HCV cases. For the HCV plasma and urine, they found 18 metabolite associations. Their research demonstrated that HCV hepatitis was consistent with impaired glucose uptake, glycolysis, and pentose phosphate pathway metabolism, with the TCA pathway fueled by branched-chain amino acids feeding gluconeogenesis and the loss of glucose from hepatocytes, which most likely contributed to hyperglycemia.

Roe et al. (2011) investigated how HCV affects the hepatocyte metabolome. They discovered that during early HCV infection, several metabolites implicated in nucleotide synthesis and RNA replication significantly increased. Along with several amino acids, NAD levels were also noticeably raised. The effects of HCV infection included altered phospholipid metabolism, possible disruptions in mitochondrial fatty acid transport, a rise in cholesterol and sphingolipid levels, and disruptions in a number of lipid metabolic pathways. Changes in the glutathione synthesis route and fluctuations in 5’-methylthioadenosine levels were also observed. Aldose reductase activity caused by AKR1B10 is elevated in hepatitis C virus infection, according to research by Semmo et al. (2015) using metabolomics. Elevated glucose, mannose, and oleamide were detected in the plasma metabolomic phenotype of HCV-positive individuals, along with decreased plasma lactate. Reduced urine excretion of fructose and galactose was present in HCV-positive individuals, while urinary excretion of 6-deoxygalactose (fructose) and the polyols sorbitol, galactitol, and xylitol was raised. These findings suggested increased aldose reductase activity, and real-time quantitative polymerase chain reaction results with increased AKR1B10 gene expression in the liver supported this conclusion. It’s interesting to note that those who had previously been infected with HCV maintained their HCV infection’s metabolomic phenotype rather than reverting to an HCV-negative phenotype, suggesting that the effects of HCV on hepatic metabolism may be long lived. A comprehensive transcriptome, proteome, and metabolome analysis of liver tissues from HCV-infected mice and hepatocyte-like cells revealed that the STAT3 signaling pathway and glucose metabolism are both increased by HCV infection, which affects speroxisome activity. Alterations in peroxisome gene expression were also connected to the outcomes of patients with liver disorders (Lupberger et al., 2019). Therefore, these metabolomics studies contribute to our understanding of the pathogenesis and development of hepatitis C and related diseases.

## Metabolomics in identifying biomarkers of hepatitis C and related diseases

The aminotransferase to platelet ratio index (APRI) and four-factor fibrosis index (FIB-4) are the most commonly used non-invasive fibrosis serologic biomarkers, both of which have high accuracy in ruling out significant fibrosis and confirming cirrhosis (Wai et al., 2003; Sterling et al., 2006). However, they were unable to identify intermediate fibrosis (Archer et al., 2022). Recently, there has been an increase in interest in metabolomics to find new biomarkers of hepatitis C, HCV-related cirrhosis, hepatic fibrosis, and HCC that are more accessible, specific, sensitive, and reproducible in a clinical context (**Table 2**).

**Table 2:** Metabolites as the diagnostic biomarkers of hepatitis C and related diseases.

The first author	Year	Cases	Sample type	Platform	Up-regulated metabolites	Down-regulated metabolites
Sarfaraz et al. (2016)	2016	fibrosis (F3-4) vs. fibrosis (F0-2)	serum	^1^H NMR	formate, 2-oxoisocaproate, methylguanidine, methionate, propionate 2-hydroxyisovalerate histidine, tyrosine, methylsuccinate caffeine, 1,7 dimethylxanthin	N-acetylaspartate, creatinine, urea, threonine, glycine, methylhistidine, adenosine, N-acetylglycine, glutamine, asparagine
Cano et al. (2017)	2017	fibrosis (F3-4) vs. fibrosis (F0-2)	serum	UHPLC-MS	sphingomyelin, phosphatidylcholine, taurochenodeoxycholic acid, taurocholic acid, tyrosine	cholesteryl ester
Khalil et al. (2022)	2022	cirrhosis vs. noncirrhosis	serum	UPLC-MS	taurocholic acid, glycholic acid, glycoursodeoxycholic acid, taurochenodeoxycholic acid, glycochenodeoxycholic acid	
Shanmuganathan et al. (2021)	2021	fibrosis (F2-4) vs. fibrosis (F0-1)	serum	^1^H NMR	choline, histidine	
Salgüero et al. (2020)	2020	cirrhosis with CTP≥7 vs. cirrhosis with CTP<7	plasma	GC-MS and LC-MS	fatty acids, bile acids, aromatic and sulfur amino acids, butyrate derivatives, oxidized phospholipids, energy- related metabolites, and bacterial fermentation-related metabolites	lysophosphatidylcholines and lysophosphatidylethanolamines, branched-chain amino acids, and metabolites of tricarboxylic acid cycle
Gaggini et al. (2019)	2019	fibrosis (F5-6) vs. fibrosis (F1-2) or fibrosis (F3-4)	serum	UHPLC/QTOF-MS	phosphocholine	ceramides, diacylglycerol
Mustafa et al. (2013)	2013	HCC vs. HCV	serum	LC-MS	arachidonyl lysolecithin, dioleoylphosphatidylcholine	uric acid, cholylglycine, 3-hydroxycapric acid, d-leucic acid, xanthine
Nomair et al. (2019)	2019	HCC vs. LC	serum	GC/MS	octanoic acid, decanoic, oleic acid, oxalic acid, glycine	
Fitian et al. (2014)	2014	HCC vs. LC	serum	GC-MS and UPLC/MS-MS	12-hydroxyeicosatetraenoic acid, 15-hydroxyeicosatetraenoic acid, sphingosine, c-glutamyl oxidative stress-associated metabolites, xanthine, amino acids serine, glycine and aspartate, a-cylcarnitines	bile acids, dicarboxylic acids
Baniasadi et al. (2013)	2013	HCC vs. cirrhotic HCV	serum	LC-MS/MS	creatine, 5-hydroxymethyl-2’ -deoxyuridine, 1-methyladenosine,	aconitic acid, 2-deoxyguanosine, glycerol, homocysteine, methionine, 1-methylguanosine, 1-methylinosine, N2,N2-dimethylguanosine, N-carbamoy, l-b-alaninephenylalanine, tyrosine, uric acid, xanthine

CTP, Child-Turcotte-Pugh; LC, liver cirrhosis; HCC, hepatocellular carcinoma; NMR, nuclear magnetic resonance; UHPLC-MS, ultra high performance liquid chromatography-mass spectrometry; UPLC-MS, ultra performance liquid chromatography-mass spectrometry; GC-MS, gas chromatography-mass spectrometry; LC-MS, liquid chromatography-mass spectrometry.

Godoy et al. (2010) reported that the metabolomics model based on NMR spectra of urine samples discriminated patients with HCV infection with high sensitivity and specificity. Additionally, the virology response of hepatitis C patients to treatment can be anticipated based on the metabolomics findings. Serum tryptophan levels in hepatitis C patients prior to treatment are considerably higher than those of patients who do not respond to treatment. These patients have a sustained virological response to the combination of PEG-IFN and RBV treatment (Saito et al., 2013).

Using NMR spectra of serum, Batista et al. (2018) developed metabolomic models to predict significant liver fibrosis, and cirrhosis in CHC in patients, which had excellent sensitivities and specificities. In addition, the models accurately classified the patients who had intermediate APRI and FIB-4 values, which might help avoid the need for these patients to have a liver biopsy. According to Salgüero et al. (2020), more advanced cirrhosis stages were characterized by an increase in plasma fatty acids, bile acids, aromatic and sulfur amino acids, butyrate derivatives, oxidized phospholipids, energy-related metabolites, and metabolites related to bacterial fermentation, while a decrease in branched-chain amino acids, TCA cycle metabolites, among other things, was observed. Lysophosphatidylcholine, taurocholic acid, and glycolic acid showed high accuracy in distinguishing patients with decompensated cirrhosis. Additionally, lower levels of sera ceramides, diacylglycerol, and phosphocholine were reported to be linked to greater levels of hepatic fibrosis and may be used as biomarkers of hepatic fibrosis (Gaggini et al., 2019). Shanmuganathan et al. (2021) observed that when compared to HCV patients with early-stage (F0-F1) liver fibrosis, serum levels of choline and histidine were reliably higher in late-stage (F2-F4) liver fibrosis patients. Using a receiver operating characteristic curve, the ratio of serum choline to uric acid allowed for the best differentiation of the severity of liver disease, and it was favorably correlated with ultrasound imaging measurements of liver stiffness. Sarfaraz et al. (2016) also demonstrated the utility of a metabolomics profiling approach to non-invasively identify biomarkers of liver fibrosis, steatosis and inflammation in patients with chronic HCV. Cnao et al (Cano et al., 2017). examined serum metabolomics in transplanted Hepatitis C patients and discovered that a model consisting of two sphingomyelins and two phosphatidylcholines accurately classifies rapid and slow fibrosers after transplantation, with an AUROC of 0.92, sensitivity of 71%, specificity of 85%, and accuracy of 84%. Fitian et al. (2014) observed the highly correlation of elevations in bile acids and dicarboxylic acids with HCV-associated cirrhosis, and these serum markers were highly sensitive and specific for cirrhosis.

Mustafa et al. (2013) conducted a study in which serum global metabolite profiles from patients with HCC and HCV were obtained using high performance liquid chromatography-mass spectrometry (HPLC-MS) techniques, making it simple to distinguish between patients with HCC and HCV and those with HCV alone. In addition, metabolomics could be applied in distinguishing HCV-related HCC from liver cirrhosis. In the study of Nomair et al. (2019), total of 34 known plasma metabolites were detected, and five of them—octanoic acid (caprylic acid), decanoic acid (capric acid), oleic acid, oxalic acid, and glycine—were found to have the strongest discriminatory potential for separating the HCC and cirrhosis groups, and ROC curve analysis revealed that oleic acid, octanoic acid and glycine had higher positive predictive value than AFP. An integrated metabolomic profiling analysis (Fitian et al., 2014) through GC/MS and UPLC/MS-MS revealed that the presence of HCV-related HCC was highly correlated with elevated levels of 12-hydroxyeicosatetraenoic acid (12-HETE), 15-HETE, sphingosine, c-glutamyl oxidative stress-associated metabolites, xanthine, amino acids serine, glycine, and aspartate, and a-cylcarnitines, and these serum markers were highly sensitive and specific for HCC. Baniasadi et al. (2013) noticed that 16 serum metabolites were substantially different between HCC and cirrhotic HCV patients. A categorization model was created and internally validated using PLS-DA analysis according to the four of the metabolites (methionine, 5-hydroxymethyl-2’-deoxyuridine, N2,N2-dimethylguanosine, and uric acid) that had the lowest p values. With sensitivity, specificity, and AUC of 97%, 95%, and 0.98, respectively, the model demonstrated excellent classification accuracy for differentiating the two groups. Furthermore, through GC-MS -based metabolomic analyses, Liu et al. (2018) suggested that the combination of glutamate and aspartate and the combination of glycerol and proline may be potential biomarkers for the prediction of HCC recurrence before and after radiofrequency ablation (RFA) treatment, respectively. According to a recent study, the fold changes in many serum bile acid concentrations showed a linear trend with hepatocellular carcinoma > cirrhosis > noncirrhosis > healthy controls, and ROC curve analysis revealed five conjugated acids, TCA, GCA, GUDCA, TCDCA, and GCDCA, that distinguished HCC from noncirrhotic liver patients (Khalil et al., 2022). Overall, metabolites in blood and urine can be used as markers to evaluate the occurrence, progression, and therapeutic efficacy of hepatitis C and related diseases.

## Metabolomics in understanding the drug action mechanism of hepatitis C and related diseases

At present, antiviral therapy is the main treatment for hepatitis C. The effectiveness of pharmacological therapy and potential biochemical mechanisms can be better understood through the use of metabolite analysis.

Direct-acting antivirals (DAAs) and ribavirin (RBV), which lead to a shorter medication period and a sustained virologic response up to 98%, are the most widely utilized antiviral treatment against the hepatitis C virus. However, a number of RBV adverse effects that are dose-related may restrict its uses (Tsubota et al., 2003). In a recent study (Giampaoliet al., 2022), RBV and its metabolites were measured in the urine of HCV patients receiving DAAs+RBV therapy in order to assess the ability of HCV patients to metabolize drugs, as well as the adverse effects such as anemia in relation to RBV metabolite levels. Some HCV patients were discovered to maintain significant levels of RBV during both the TW4 and EOT stages, while another several patients were discovered to maintain high levels of RBV proactive metabolites, most likely because of nucleosidase activity. Hepatic fibrosis and metabolic changes are caused by oxidative stress and a protracted inflammatory response brought on by HCV infection in the liver microenvironment. Liver damage is only partially repaired even though DAAs cause HCV-clearance. According to the study of Biliotti et al. (2021),a catabolic intermediate of nicotinamide-adenine-dinucleotide, 1-methylnicotinamide, was considerably elevated in HCV patients and restored following HCV clearance, most likely as a result of the diminished hepatic inflammation. An enhancement in skeletal muscle protein synthesis was suggested by 3-hydroxy-3-methylbutyrate, a leucine-catabolism intermediary that was completely restored following HCV elimination. Glycine and choline momentarily rose throughout therapy, as did 3-hydroxyisobutyrate and 2, 3-dihydroxy-2-methylbutyrate, intermediates of valine catabolism. Using LC-MS/MS and lipoprotein electrophoresis assays, Chang et al. (2018) demonstrated that at 24 weeks post-anti-HCV-therapy, accelerated cholesterol biosynthesis, hepatic lipid export, ω-oxidation and decreased systemic inflammation were noted in CHC patients with sustained virological response (SVR); while HCV-associated lipid metabolic alterations required >24 weeks for restoration or were incompletely reversible after SVR. Jun et al (Wang et al., 2013). showed the effectiveness of metabolomics in capturing and elucidating metabolic characteristics of HCV and the therapeutic benefits of the bear bile powder (PBBP). Keogh et al. (2017) conducted a GCMS-based metabolomic investigation to clarify the mechanisms underlying the efficacy of a novel anti-Hepatitis C virus and anti proliferative agent in altering metabolic networks in hepG2 and hep3B Cells, which showed that metabolomics can provide mechanistic insights into the efficacy of novel drug candidates prior to the appearance of their pharmacological effect. Therefore, metabolomics is an effective method to explore the mechanism of drug treatment of hepatitis C and related diseases.

## Metabolomics in hepatitis E and related diseases

HEV is a significant zoonotic virus that can affect a variety of hosts. It is mainly transmitted through the fecal-oral route and has obvious seasonality. It has eight major genotypes. Most HEV-infected individuals have no symptoms, but some may have jaundice and other acute hepatitis symptoms (Wu et al., 2019; Wu and Huang, 2020). HEV infection can also lead to a variety of extrahepatic symptoms (Wu et al., 2021). Although HEV infection typically results in acute and self-limiting diseases, acute liver failure (ALF) brought on by HEV infection has increased in prevalence over the past few years, with serious implications for patient outcomes (Wu et al., 2020).

Using ultra-performance liquid chromatography-mass spectroscopy (UPLC-MS), a recent study showed that there were significant differences in the serum metabolite components between AHE patients and healthy controls, or between HEV-ALF and acute hepatitis E (AHE) patients. Their research indicated that dynamic changes in serum metabolites were related to the occurrence and severity of AHE, and can be used as diagnostic and prognostic markers for HEV-ALF (Wu et al., 2022).

Munshi et al. (2011) discovered variations in the concentrations of several metabolites in the plasma and urine of hepatitis E patients. According to pathway analysis methods, glycolysis, urea cycle, TCA cycle, and amino acid metabolism may be involved in acute hepatitis E patients. These discoveries could contribute to a better understanding of the pathophysiologic mechanisms at play in this disease as well as its clinical and biochemical manifestations. Currently, researches on metabolomics in hepatitis E and related diseases are still scarce, and the in-depth mechanism discussion and markers studies may be the hot topics in the future.

## Limitations and perspectives

Metabolomics is still a developing field, and as such, it still has several limitations, like the variety of detection samples. Emphasis must be placed on the value of reliable metabolite measurement and standardization of biological materials. The type and quantity of metabolites can also be affected by a variety of variables, including sampling time, collection method, treatment, storage stability, extraction, sample dilution, measurement methods, and statistical analysis. In addition, outside variables like body mass index, diet, physical activity, and treatment may also have an impact on the progression of the disease and consequently metabolite levels.

The use of metabolomics offers a fresh approach and viewpoint for understanding the mechanisms of occurrence and development, diagnosis, prognosis, and therapy of viral hepatitis and related diseases. The metabolomics of HBV, HCV, and associated disorders have been extensively studied. Further associations have emerged and several biomarkers have been discovered. The efficacy of these screening indicators in patient diagnosis and prognosis, however, is still up for debate. Furthermore, the metabolomics of other hepatitis is insufficient and needs further investigation.

## Author contributions

All the authors contributed substantially to this review. YW: conceptualization and design. JY, DW and YL: manuscript drafting. HW and QH: revising, checking, and approving of the final version of the manuscript.
